# Supplementary material for: The interactome of intact mitochondria by cross-linking mass spectrometry provides evidence for coexisting respiratory supercomplexes
Source: Mol Cell Proteomics. 2017 Dec 8;17(2):216–32. doi: 10.1074/mcp.RA117.000470 (PMC5795388; doi:10.1074/mcp.RA117.000470)
Supplement: Supplemental Data [file supp_RA117.000470_133922_0_supp_23978_fzffwf.zip › spectra_annotation/mito_DR_spectra_annotation/99-1-40-1-15-1.pdf]

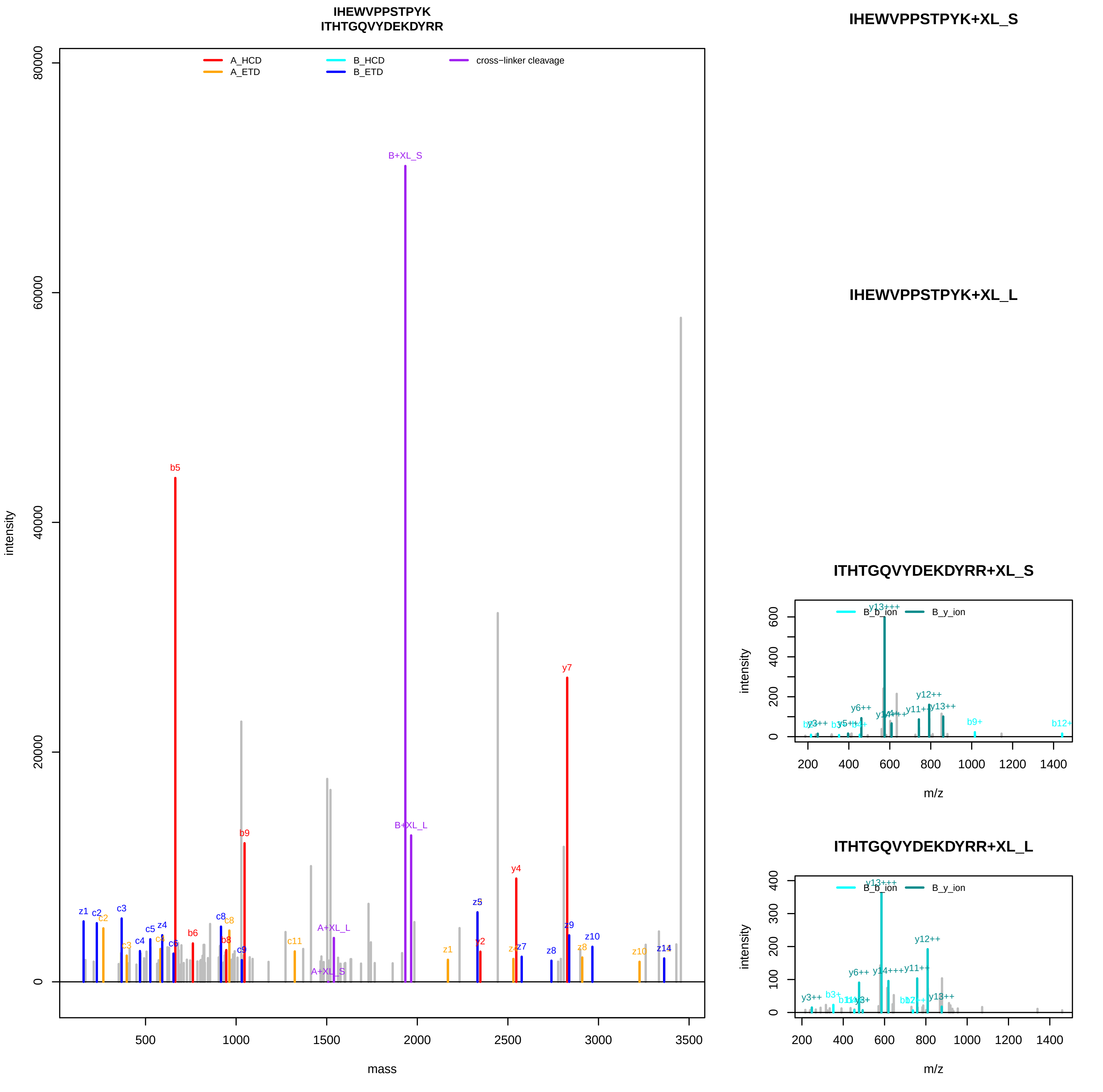
IHEWVPPSTPYK
ITHTGQVYDEKDYRR
A_HCD
A_ETD
B_HCD
B_ETD
cross−linker cleavage
B+XL_S
B+XL_L
A+XL_L
A+XL_S
b5
b6
b8
b9
y2
y4
y7
z1
c2
c3
c4
c5
z4
c6
c8
c9
z5
z7
z8
z9
z10
z14
c11
z1
z8
z10
intensity
mass
0
20000
40000
60000
80000
500
1000
1500
2000
2500
3000
3500
IHEWVPPSTPYK+XL_S
IHEWVPPSTPYK+XL_L
ITHTGQVYDEKDYRR+XL_S
B_b_ion
B_y_ion
y13+++
y12++
y11++
y13++
y6++
y5++
y3++
b9+
b12+
intensity
m/z
0
200
400
600
800
1000
1200
1400
ITHTGQVYDEKDYRR+XL_L
B_b_ion
B_y_ion
y13+++
y12++
y11++
y14+++
y6++
y3++
b3+
y13++
intensity
m/z
0
100
200
300
400
